# A shifting paradigm in the aetiology of oral and pharyngeal cancer in Sri Lanka: a case-control study providing serologic evidence for the role of oncogenic HPV types 16 and 18

**DOI:** 10.1186/s13027-015-0007-z

**Published:** 2015-04-16

**Authors:** Suvanthee Kushani Gunasekera, Kanthi Angela Perera, Chandrika Fernando, Preethi Vidya Udagama

**Affiliations:** Department of Zoology, Faculty of Science, University of Colombo, Colombo 03, Sri Lanka; National Cancer Institute, Maharagama, Sri Lanka; Sri Lanka Institute for Information Technology, Colombo, Sri Lanka

**Keywords:** Sri Lanka, Oral and pharyngeal cancer, Human papillomavirus (HPV), HPV16, HPV18, Enzyme-linked Immunosorbent assay, Risk factors, Smoking

## Abstract

**Background:**

Oral and pharyngeal cancer (OPC) of multifactorial aetiology is a major health problem globally. Ranking first in all cancers, OPC poses a significant impact on the Sri Lankan male population. As Human Papillomavirus (HPV) high risk (HR) types are found to be significant risk factors for OPC globally, the current study was undertaken to examine the association between HR-HPV16 and 18 types with OPC in Sri Lanka.

**Materials and methods:**

Serum samples of 78 OPC patients and 51 non-cancer controls were assayed for the presence of anti-HPV16 and anti-HPV18 IgG antibodies using in-house established Enzyme Linked Immunosorbent Assays (ELISAs). The association between OPC and its risk factors *i.e.* HPV, smoking, alcohol, betel quid, poor dentition, was established using Chi-square test. Logistic regression was used to calculate odds ratios (OR), adjusted for the influence of other risk factors.

**Results:**

This prototype study in Sri Lanka showed a significant risk of 15 fold in developing OPC due to HPV16/18 seropositivity after removing variability due to other factors. Oncogenic HPV18 showed a higher rate of seropositivity being detected in 32% of OPC patients, and also in 2% of non-cancer control subjects. HR-HPV16 was detected in 23% of OPC patients and in 5.88% of controls. Moreover, seven OPC patients were detected with both anti-HPV16 and anti-HPV18 antibodies. According to the logistic regression models HPV18 seropositivity was associated with a 28 fold risk in developing OPC while that of HPV16 was associated with a 6 fold increase in risk for the development of OPC. A 5 fold risk of developing OPC was also pronounced among smokers while alcohol, betel and poor dentition was not significantly associated with OPC. Statistically significant differences with regard to age, gender, smoking, alcohol, betel use, poor dentition and site specificity of the tumour was not observed between HPV seropositive and seronegative OPC patients.

**Conclusions:**

Both in-house developed ELISAs detected significant proportions of HPV seropositives within the OPC study population suggestive of HPV as a strong risk factor for oral and pharyngeal carcinogenesis in Sri Lanka.

## Background

Oral and pharyngeal cancer (OPC) is a significant component of the global burden of cancer, worldwide being the sixth most common cancer and the eighth most common cause of cancer death [1,2]. Sri Lanka is a high risk region for the disease with high incidence rates globally [3]. Also, collectively lip, oral cavity and pharyngeal cancer rank first in cancer incidence in the male population of Sri Lanka (Cumulative rate 1.906) with increasing age specific rates [4].

Several epidemiologic investigations carried out globally documented the correlation between consumption of tobacco, alcohol, and betel chewing as major risk factors for developing OPC with a dose-response relationship [5-7].

In developing countries up to 23% of malignancies are caused by infectious agents, including HPV [1]. HPV, in addition for being a successful sexually transmitted infectious agent associated with anogenital warts and mild dysplasia [8] has cell-immortalizing and cell-transforming properties [9] which mediate oncogenesis. The high-risk human papillomavirus (HR-HPV) types are well confirmed as aetiologic agents of cervical cancer [10,11]. Compelling studies carried out worldwide provide sufficient evidence that infection with HR-HPV can be implicated in the pathogenesis of OPC [12-15].

Presence of HPV DNA in a sample population of Sri Lankan patients with oral squamous cell carcinoma was previously reported. The positivity was detected by PCR and direct cycle sequencing and the highest HPV positivity was found in the buccal mucosa. The overall HPV prevalence of the study sample was 37.2% (N = 102) [16]. Thus, apart from betel quid the study suggested that HPV is a strong risk factor for oral carcinogenesis in Sri Lanka.

The current study examined the associations between established risk factors of OPC, with special emphasis on HPV as a suspected major emerging risk factor determining oral and especially pharyngeal cancer in Sri Lanka. Sero-prevalence of HPV was detected with in-house developed indirect enzyme-linked immunosorbent assays (ELISAs) specific for HPV16 and HPV18, using virus-like particles (VLPs) produced for the L1 region of the HPV genome.

## Results

### Study subjects: Socio-demographic factors and medical information

Cases (N = 78) and controls (51) were comparable with regard to age and gender (P > 0.05). Males (94%) were more likely than females (6%) to be diagnosed with OPC. Age of cases ranged from 38 to 77 years. A higher proportion of the patients (66.7%) had only received primary education. From the 25 districts in Sri Lanka, residency of the cases covered ten.

Among the cases, 33 (42%) had cancers of the oral cavity while 45 (58%) had pharyngeal cancers with sub sites including floor of the mouth, hard palate, tongue, retro molar buccal mucosa, soft palate, tonsil, cheek, maxilla antrum and oropharynx. Patients were not diagnosed for any other carcinomas previously with the exception of one case with a cancer in the sigmoid colon, and a majority (86%) had no family history of cancer.

### Anti-HPV IgG detection and HPV as a risk factor for OPC

**Figure 1:**
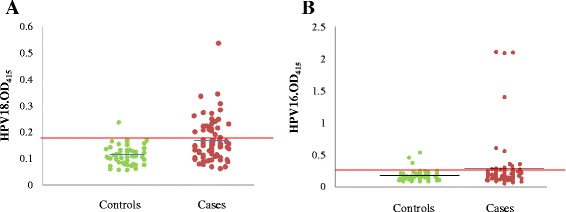
Anti-HPV IgG responses in OPC patients and non-cancer controls in a Sri Lankan population. (**A**) Anti-HPV18L1 and (**B**) Anti-HPV16L1 IgG responses of the case and control groups. Each enclosed circle represents the mean of duplicate OD values at 415 nm, of each serum sample. Long horizontal lines represent the cut-off values (the mean OD value at 415 nm plus 2 standard deviations of the control group excluding outliers) and the short horizontal lines represent the mean values for HPV18L1 and HPV16L1 ELISA. Mean OD values obtained for test samples falling over and above this cut-off level were expressed as positive responses.

Serologic response, *i.e*. anti-HPV16/18 IgG antibodies, of the case and control subjects is shown in Figure 1. The cut-off values for HPV16 L1 and HPV18 L1 indirect ELISAs estimated after the exclusion of outliers in the control group were 0.262 and 0.1777, respectively (Figure 1). The mean OD values for anti-HPV IgG response of the case group were significantly higher than that of the control group for both HPV16 and HPV18 VLPs (HPV18: P = 0.00; HPV16: P = 0.035).

Seropositivity to HPV18 L1 VLP was significantly different between cases (32%) and controls (1.96%). The seropositives pose a momentous 24-fold increase in risk for OPC when compared with seronegatives (OR = 23.57; 95% CI: 3.08-180.62). Although, the wide confidence interval indicates an imprecise estimate due to the small sample size, the magnitude of risk estimate suggests a strong positive association between seropositivity to HPV18 and OPC. A significant difference (P = 0.019) was also detected in the seropositivity of HPV16 cases (18/78; 23.1%) compared with the controls (3/51; 5.8%), but with only a 4.8-fold risk in developing OPC with reference to the seronegatives (OR = 4.8; 95%CI, 1.335-17.261). OPC patients seropositive for either HPV16 or HPV18 comprised 46% of the cases with a significant difference with the seropositivity of the control group (P = 0.00), and with a 13.71-fold risk of developing OPC with contrast to seronegatives (Table 1). Seven cases were co-infected with HPV16 and HPV18.

**Table 1:** **Association of OPC with prevalence of anti-HPV IgG antibodies in a Sri Lankan study population**

**Characteristic**	**OPC cases (N = 78)**	**Controls (N = 51)**	**Chi-square**	**P** ***-*** **value**	**Unadjusted odds ratio (95% CI)**
**HPV16/18 L1 serologic status**					
**Seronegative**	42 (53.8%)	48 (94.12%)			1.0
**Seropositive**	36 (46%)	3 (5.88)	21.84	0.00	13.714(3.935-7.795)
**HPV16L1 serologic status**					
**Seronegative**	60 (76.9%)	48 (94.1%)			1.0
**Seropositive**	18 (23.1%)	3 (5.8%)	5.487	0.019	4.8 (1.335-17.261)
**HPV18 L1 serologic status**					
**Seronegative**	53 (68%)	50 (98.04%)			1.0
**Seropositive**	25 (32%)	1 (1.96%)	15.531	0.00	23.56(3.08-180.620)

**Figure 2:**
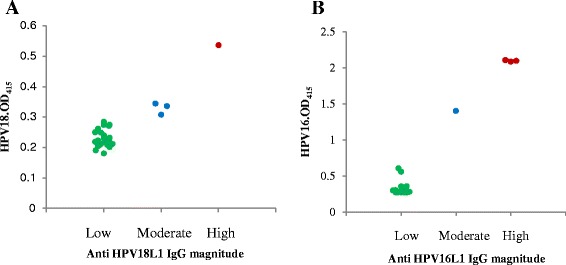
Type-specific distribution of anti-HPVL1 IgG magnitudes of the HPV seropositive oral and pharyngeal cancer patients. (**A**) HPV18 L1 and (**B**) HPV16 L1. Each circle represents a single sample. Anti-HPV18L1 IgG magnitudes: Low (OD_415_< 0.3), Moderate (0.3 < OD_415_< 0.5), High (OD_415_> 0.5). Anti-HPV16L1 IgG magnitudes: Low (OD_415_< 1.0), Moderate (1.0 < OD_415_< 2.0), High (OD_415_> 2.0).

Results of each indirect ELISA revealed 3 groups for anti-HPV IgG positivity (Figure 2). Based on the assumption that the OD value is directly proportional to the antibody magnitude and hence to the viral load, the groups can be defined as being low, moderate and high. In addition, cases detected with ‘high’ and ‘moderate’ anti-HPV antibody magnitudes had stage IV tumours. Besides, seropositive individuals for anti-HPV16 antibodies showed higher OD_415_ values (ranging from 0.26 to 2.1) than those with anti HPV18 antibodies (0.177 to 0.536), indicative of a higher antibody response for HPV16 compared with HPV18. This was reiterated for OPC patients with co-infections of HPV16 and HPV18, where a majority presented with higher OD values for HPV16.

### Other risk factors associated with oral and pharyngeal cancer

The association of OPC with smoking, alcohol, betel quid chewing and poor dentition is shown in Table 2. Poor dentition (manifested as tooth loss) was excluded as a significant risk factor for OPC in this study as there was no significant difference (P = 0.213) observed between cases and controls (Table 2). Among the smokers, 50% (39/78) were light smokers (<10 cigarettes/day), 20.5% (16/78) were moderate smokers (10-20 cigarettes/day) while 11.5% (9/78) were heavy smokers (>20 cigarettes/day). Comparing smoking intensity with the number of years smoked, 82.5% (52/63) of the smokers had been exposed to tobacco for more than 20 years and in 25.4% (16/63) the usage time exceeded 40 years. Of the heavy smokers the majority had a usage time period of 15-30 years while only 1 case was a smoker for 50 years.

**Table 2:** **Association of OPC with well-established risk factors: smoking, alcohol, betel and poor dentition**

**Characteristic**	**OPC cases (N = 78)**	**Controls (N = 51)**	**P** ***-*** **value**	**Unadjusted odds ratio (95% CI)**
**Smoking**				
**Smokers**	63 (80.8%)	23 (45.1%)		5.113 (2.325-11.246)
**Non-smokers**	15 (19.2%)	28 (54.9%)	0.000	1.0
**Alcohol**				
**Users**	70 (89.7%)	37 (72.5%)		3.311(1.273-8.609)
**Non-users**	8 (10.3%)	14(27.5%)	0.021	1.0
**Betel**				
**Users**	51 (65.4%)	21 (41.2%)	0.012	2.698 (1.304-5.583)
**Non-users**	27 (34.6%)	30 (58.8%)		1.0
**Poor dentition**				
**Tooth loss**	54 (69.2%)	29 (56.8%)	0.213	1.707 (0.820-3.555)
**No loss**	24 (30.8%)	22 (43.1%)		1.0

### Aetiology of OPC with independent risk assessment

Influence of each risk factor for the development of OPC after adjusting for the influence of other significant risk factors using binary logistic regression models is shown in Table 3.

**Table 3:** **Risk of developing OPC due to established and emerging aetiological factors in Sri Lanka**

**Risk factor**	**Model 1** ^**a**^		**Model 2** ^**b**^		**Model 3** ^**c**^		**Model 4** ^**d**^	
	**P-value**	**OR (95% CI)**	**P-value**	**OR (95% CI)**	**P-value**	**OR (95% CI)**	**P-value**	**OR (95% CI)**
**HPV 18**	_	_	0.002	28.29 (3.444-232.412)	0.005	20.78 (2.56-168.80)	0.00	15.15 (3.933-58.346)
**HPV16**	0.012	5.89 (1.466-23.676)	_	_	0.088	3.49 (0.829-14.683)		
**Smoking**	0.001	4.65 (1.805-11.957)	0.002	4.94 (1.769-13.809)	0.003	4.92(1.732-13.95)	0.004	4.67(1.658-13.161)
**Alcohol use**	0.645	1.33 (0.395-4.475)	0.734	1.26(0.326-4.904)	0.622	1.42(0.354-5.67)	0.525	1.56(0.399-6.075)
**Betel quid use**	0.114	1.92(0.855-4.306)	0.127	1.95(0.827-4.576)	0.204	1.76(0.735-4.222)	0.320	1.56(0.650-3.737)

^a^ **Model 1** Adjusted for HPV16, smoking, alcohol and betel.

^b^ **Model 2** Adjusted for HPV18, smoking, alcohol and betel.

^c^ **Model 3** Adjusted for HPV18, HPV16, smoking, alcohol and betel.

^d^ **Model 4** Adjusted for HPV16/18, smoking, alcohol and betel.

In the first model, after accounting for other factors (smoking, alcohol consumption and betel quid chewing) cases being seropositive for HPV16 was statistically significant with reference to the control subjects (P = 0.012) with a risk of 5.89-fold. A case subject being seropositive for HPV18 was statistically significant with reference to the control subjects (P = 0.003) after adjusting for smoking, alcohol consumption and betel quid chewing. The seropositives showed a marked increase of a 28-fold risk in developing OPC compared with the seronegatives (OR = 28.29) (model 2). The adjusted OR for both HPV16 and HPV18 was higher than the unadjusted OR suggesting that HPV16 and HPV18 unaided act as an important risk factor for OPC which can drive an oncogenic effect notwithstanding other risk factors. As per the third logistic regression model only HPV18 and smoking posed significant risks. Individuals seropositive for HPV18 were associated with a 20-fold risk of developing OPC after removing influence due to other factors. Smoking accounted for risk of 4.92 fold. Those who were seropositive with either high-risk HPV16 L1 or HPV18 L1 or both, showed a 15-fold risk of developing OPC relative to seronegative subjects for the two HPV types after controlling for other risk factors (model 4). Accordingly, smoking was also found to be significant (P = 0.004) with a 4.67-fold risk factor. Alcohol use and betel quid chewing were not statistically significant (P > 0.05) risk factors in developing OPC when exposure to HPV18 and/or HPV16 was present. Conversely, smoking was a significant risk factor in all models developed even after adjusting for HPV seropositivity.

None of the established risk factors such as smoking, alcohol use, betel quid chewing, poor dentition nor age and gender (data not shown), were significantly different (P > 0.05) between the HPV16/18 seropositive and seronegative OPC patients.

### HPV seropositivity and associated factors

**Figure 3:**
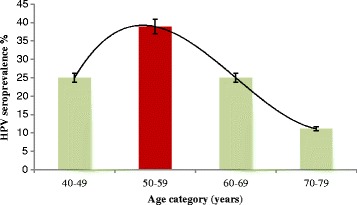
Age distribution of HR-HPV16 and/or HR-HPV18 seropositivity, in 36 oral and pharyngeal cancer patients from Sri Lanka.

Of the OPC patients HPV seropositivity was detected in individuals ranging in age from 41-72 years of which a majority (38.8%) was within the 50-59 age group (Figure 3). HPV seropositivity of the 40-49 and the 60-69 groups were similar (25%) with a decline thereafter. Male predominance was observed with HPV seropositive OPC patients at a ratio of 17:1.

Seropositivity of HPV was higher among light smokers (41.6%) and non-smokers (25%). Notably three of the seven patients co-infected with HPV16 and 18 types were non-smokers. The anti-HPV antibody magnitudes of these three cases were ‘high’ for HPV16 while high antibody magnitude for HPV18 was detected in a single patient.

Patients with cancers of both the oral cavity and the pharynx were detected with anti-HPV16L1/18 L1 IgG antibodies. A higher seropositivity for anti-HPV antibodies were detected in patients with pharyngeal than those with oral cancer. None of the tumour characteristics (tumour grade, size, stage and nodal involvement) showed a significant difference (P > 0.05) with the HPV serologic status (Table 4). Remarkably, 27.8% of HPV seropositives were diagnosed with cancers of the tonsils. Of the seven OPC patients co-infected with HPV16 and HPV18, 4 were oral cancer patients and 3 were diagnosed with pharyngeal cancer. Three patients did not show any nodular involvement and all 7 cases were at an advanced tumour stage (Stage III:2; Stage IV:5). The tumour grade of all 7 cases were either moderately or well differentiated. Influence of other risk factors and histopathologic characteristics on the dual exposure could not be assessed statistically due to the small sample size.

**Table 4:** **Association of tumour characteristics with HPV seropositivity in OPC-patients of a Sri Lankan study population**

**Characteristic**	**HPV**		**χ** ^**2**^ **value**	**P-value**
	**Negative**	**Positive**		
**Tumour site (N = 78)**				
**Oral cavity**	20	13	0.633	0.426
**Pharynx**	22	23		
**Tumour grade (N = 55)**				
**Poor/undifferentiated**	1	1		
**Well/moderate**	26	27	0.00	1.00
**Tumour size (N = 61)**				
**T0-T2**	12	17		
**T3-T4**	16	16	0.174	0.676
**Nodal involvement (N = 61)**				
**Yes**	24	25		
**No**	4	8	0.425	0.515
**Stage (N = 60)**				
**I/II**	2	4		
**III/IV**	25	29	0.03	0.863

## Discussion

Sri Lanka is facing a precarious health problem with oral and pharyngeal cancer (OPC) having the highest age standardized rate per 100,000 populations (ASR/P: 15.5) for oral cancer in Asia and ranking 3^rd^ in the world. For pharyngeal cancers Sri Lanka ranks 4^th^ in Asia and 11^th^ in the world with an ASR/P of 7.2. Notably, both exceed the world ASR/P [3].

There is sufficient evidence for a causal role of tobacco, alcohol, betel quid chewing and poor oral hygiene with OPC in Sri Lanka [17-20], but lacked association with HPV. Albeit, prevalence of HR-HPV in females diagnosed with cervical cancer has been studied [21], reports on HPV burden on men and OPC is scarce. However, a study investigated HPV in oral but not pharyngeal cancers in Sri Lanka [16]. Therefore, detection of anti-HR-HPV antibodies in patients of pharyngeal cancer in the present study provides novel insights to the risks of OPC in Sri Lanka.

The infection with HR-HPV typically lasts from 12-18 months and is eventually cleared by the immune system [22]. Compared with HPV infections that clear, persistent HR-HPV infections have a higher chance of progressing in to malignancy [23] that usually takes a course of more than 10 years [24]. Many studies have concluded that the presence of anti-HPV antibodies are correlated with oral and pharyngeal tumors that were positive for HPV DNA compared to HPV DNA negative tumors [25-27]. The current study substantiated the association of HPV16 and HPV18 with the aetiology of OPC in Sri Lanka, evidenced by the detection of anti-HPV16 and anti-HPV18 IgG antibodies detected by in-house developed ELISAs.

Global prevalence and type distribution of HPV in OPC remains unclear [28]. The striking 28-fold risk obtained from model 2 (Table 3) evidenced that HPV18 may be a major risk factor for the development of OPC in Sri Lanka. Furthermore, the high magnitude of the OR for HPV18 seropositivity increased after controlling for the confounding effect of smoking, alcohol and betel chewing indicating HPV18 as an independent risk factor for OPC. These results agree with a study investigating HPV in oral squamous cell carcinoma in a Brazilian population where they found HPV18 as the most prevalent HPV type in their study population [29]. Conversely, in the present study, HPV16 posed only a 4.8-fold risk of developing OPC which increased after adjustment for smoking, alcohol and betel chewing.

Nonetheless, this indicates a paradox with most of the prevailing records on HPV related OPC. A multicenter study conducted in nine countries evidenced that HPV16 unaided accounted for more than 90% of HPV-positive OPC, and that HPV18 was less of an important risk factor as this was detected infrequently in OPC patients [12]. However, studies conducted in Taiwan revealed that HPV16 and HPV18 had co-dominant roles in the aetiology of oral carcinogenesis [30,31].

The risk of malignancy and differential response towards OPC in individuals who are exposed to more than one type of HR-HPV is still unclear [32]. However, approximately 20% of HPV infected OPC patients in the current study were detected with both anti-HPV16 and anti-HPV18 antibodies. Influence of other risk factors and histopathologic characteristics on the dual exposure could not be assessed statistically due to the small sample size of the study. Nevertheless, it can be concluded that HPV16 and HPV18, independently or in synergy are associated with risk of carcinogenesis in the oral and pharyngeal region.

The aetiology of an infectious disease is dependent on the agent, host and environment [33]. However, viral oncoproteins of HR-HPV types are implicated as the drivers of transformation in HPV related OPC and evidence suggests that HR-HPV strains may initiate oral carcinogenesis among abstainers of alcohol and tobacco [34,35]. Thus, investigating the life style factors associated with OPC patients are important in drawing suppositions for the involvement of HPV in OPC. Smoking was confirmed as a strong risk factor for OPC. Moreover, the decrease in the association after adjustment for other risk factors (alcohol, betel and HPV16/18) was negligible denoting that smoking contributes substantial independent risk prediction. Alcohol consumption is found to be associated with increased risk for OPC, with dose-response relationships observed [36-38]. Betel chewing is a major risk factor for the development of OPC especially in the Asian region [39]. In a study conducted in Sri Lanka, 64% of oral cancer patients were habitual betel chewers [16]. Betel chewing is still practiced in both rural and urban areas of the island with new forms (‘beeda’) being introduced [20]. Conversely, the current study excluded the significance of both alcohol consumption and betel quid chewing as risk factors for OPC in the presence of HPV16/HPV18 infection.

That HPV-seropositive OPC was not significantly associated with age of the patient in the current study, contradicts the postulate that younger age groups (30-50 years) are at a higher risk of developing OPC due to HPV [40]. Since infection with HPV is likely an early oncogenic event in OPC, the rise of HPV seropositives in the age group of 50-59 years may be an indicator for the infection occurring in the 4^th^ decade of their life. The Sri Lankan situation may be different from that of other geographical regions having middle aged men more inclined to the risk, though the small sample size of the present study may pose a limitation on drawing presumptions.

Studies indicate that HPV plays a definite aetiologic role in cancers of the oropharynx and in a small group of cancers of the oral cavity [12,15]. The current study showed no significant difference in HPV16/18 seropositivity for cancers in the oral cavity and the pharynx. Though not significant (P > 0.05) probably due to the small sample size, a 4 fold increased risk with alcohol and 2 fold increased risk with HPV18 seropositivity and smoking was associated with pharyngeal cancer.

In general, at the base-line, tumor stage III/IV, poorly differentiated tumor grade and N2-3 nodal involvement show significantly increased risk in detecting anti-HPV antibodies [41]. Due to lack of awareness and education, early stages of OPC are not detected among Sri Lankan patients at the time of diagnosis. Thus, such correlations could not be inferred from the current study.

The increasing prevalence rate of HPV in OPC highlights the change of life style in sexual behavior. The route of HPV infection in the oral and pharyngeal region is not firmly established [27]. However, epidemiologic evidence suggests sexual transmission, though direct mouth-to-mouth contact or other means are not ruled out [13]. Social and cultural barriers in Sri Lanka, failed the effort of investigating the mechanism of transmission of the virus by assessing the sexual history of the participants in this study.

The current study suggests four aetiologic categories of OPC in Sri Lanka: Low risk HPV positive tumors arising in non-smokers; moderate risk HPV positive tumors arising in former or current smokers; high risk HPV-negative tumors arising in current or former smokers; and the fourth category being HPV negative tumors arising in non-smokers. The fourth category may include heavy alcohol use, habit of betel chewing or any other variant associated with OPC.

A major limitation of this research is the case-control design of the study which precludes establishment of a causal link between HR-HPV and OPC. As serological response to HPV is a surrogate marker of both current and past infection, it does not indicate whether infection was acquired prior to the development of OPC. An OPC patient could have acquired HPV infection few months prior to being diagnosed with OPC and hence had positive serology at the study enrollment, wherein HPV would not be ‘causally’ associated with OPC. Hence, a prospective longitudinal study should be undertaken to establish causal relationship between HPV and OPC.

Interestingly, HPV positive tumors respond well to treatment showing a potential benefit of a more favorable outcome from therapy compared to HPV negative OPC [42]. Thereby, the variation in response to therapy has been of consideration for de-escalation of treatment intensity for these tumors [43] and retrospective data show that HPV positive OPC are readily cured using radiotherapy alone [44]. Thus, HPV status of the OPC patient at diagnosis can be a predicting factor in determining the course of treatment. Since, 22% of HPV positive cases were non-smokers, the outcome of the current study may be an incentive for prospective epidemiologic studies using larger sample sizes to change the face of therapy in OPC in Sri Lanka. De-escalation of therapy will reduce the morbidity of patients and importantly will be less costly, that would benefit a developing country such as Sri Lanka that provides free healthcare service to the public. Knowledge on HPV serologic status may also be a predicting variable for the development of OPC in asymptomatic individuals where early treatment measures can be implicated. Future investigations involving molecular evidence *i.e* analysis of HPV DNA in formalin fixed paraffin embedded cancer tissues and expression of its oncogenic genes E6 and E7, to support the role of HPV in the pathogenesis of oral and pharyngeal cancer, and epidemiologic evidence that will guide HPV related oral and pharyngeal cancer control and prevention programmes in Sri Lanka will be of great importance in the local and global scale.

## Conclusions

This study was the first to follow established major risk factors with HR-HPV in both oral and pharyngeal carcinoma in Sri Lanka. Two in-house established ELISAs detected anti-HPV16 and anti-HPV18 IgG antibodies. The results showed a plausible association between HR-HPV and OPC with 46% of the case group being seropositive for HPV16/18; HPV18 and HPV16 were detected in 32% of the patients with a risk of 28 fold and in 23% with a 6 fold risk confounding other risk factors, respectively. Smoking was significantly associated with OPC posing a 5 fold risk.

## Methods

### Ethical approval

The case-control study received approval of the Ethics Review Committee, Faculty of Medicine, University of Colombo (EC12/29), Sri Lanka.

### Study subjects and data collection

Clinically diagnosed OPC cases were recruited from the National Cancer Institute, Sri Lanka between April-September 2012. Newly enrolled patients and those who received a single dose of chemotherapy or radiotherapy for OPC were the inclusion criteria. Exclusion criteria were severely ill patients and individuals who declined to participate. Written, informed consent was obtained prior to the interview and sample collection. Patients were interviewed using an approved questionnaire to accrue information on socio-demographics, use of tobacco, alcohol and betel, oral hygiene and sexual practices. Medical information of the patients was retrieved from their clinical records. Comparable to the enrolled cases (N = 78), 51 age and gender matched healthy individuals with no personal history of cancer were recruited as controls to the study.

Variables on smoking history were ascertained by general discussion that assessed the number of cigarettes smoked per day, age at which an individual started to smoke, the number of years smoking was practiced and number of years since quitting. Similar information was obtained regarding lifetime consumption of alcohol and betel quid.

A supplemental question included in the questionnaire, which inquired on sexual practices, was not pragmatic due to social and cultural barriers in Sri Lanka.

### Collection of serum samples

A sample of blood (1 ml) was aseptically collected from the study subjects via intravenous bleeding. Separated serum samples were stored at -20°C until further use [45].

### Anti-HPV IgG detection using Indirect Enzyme-linked Immunosorbent Assay

IgG antibodies against HPV-specific proteins were assayed using two in-house established indirect enzyme-linked immunosorbent assays (ELISA).

HPV virus-like particles (VLP) for HPV types 16 and 18 expressing L1 protein and H16.V5 and H18.J4 type-specific monoclonal antibodies [46] were used to determine optimal antigen concentrations. Reagents and buffers were optimized using checker board titrations.

Wells of microtiter plates (Immulon2, Germany) were coated with 100 μl of VLPs (HPV16 VLP: 4.6 μg/ml, HPV18 VLP: 4 μg/ml) in PBS (pH 7.4) and incubated overnight at 4°C. All subsequent incubation steps involved 37°C for 1 hour. Non-specific binding sites were blocked with milk buffer (5% w/v non-fat milk powder in PBS) and the plate was flick-washed with washing buffer (0.5% Tween20 in PBS). Human serum diluted in dilution buffer (5% w/v non-fat milk powder in washing buffer) at 1:100 was added to duplicate wells and following incubation, unbound reagents were removed by flick-washing. The bound antibodies were detected with alkaline phosphatase linked anti-human IgG antibody (Sigma chemicals, USA) and the reaction was visualized by the addition of PNPP (HIMEDIA, India). The reaction was arrested with 3 M NaOH and the optical density (OD) was measured at 415 nm in an automated ELISA plate reader (Model 680, BioRad International, USA).

The negative control method [47] was used to determine the cut-off value (COV) for the HPV seropositivity. The outliers of the control group were excluded when setting the cut-off values. The COVs for the two ELISAs were derived by using the mean OD value of the negative controls plus 2 times the standard deviation.

### Statistical analyses

Statistical analyses were performed using SPSS version 16 for Windows (SPSS Inc, USA) software package. Comparisons of normally distributed variables of independent samples were performed using the t-test. When variable distribution was not normalized, the Mann-Whitney U test was used to compare variables between groups. The Chi-square test was used to compare categorical variables between groups (e.g. cases vs. controls, seropositive cases vs. seronegative cases). Binary logistic regression was used to model the relationships between presence of OPC and its predictor variables. Calculated odds ratio (OR) measured the risk of developing OPC. Wald statistic was used to determine parameter significance (P-value) in logistic regression analyses. The level of significance was set at P < 0.05.

## Abbreviations

OPC: Oral and pharyngeal cancer
HR: High risk
HPV: Human papillomavirus
ELISA: Enzyme-linked immunosorbent assay
OR: Odds ratio
VLP: Virus-like particle
OD: Optical density
COV: Cut-off value
ASR/P: Age standardized rate per 100 000 population
